# Prognostic Value and Potential Role of Alternative mRNA Splicing Events in Cervical Cancer

**DOI:** 10.3389/fgene.2020.00726

**Published:** 2020-07-10

**Authors:** Xiang-yang Shao, Jin Dong, Han Zhang, Ying-song Wu, Lei Zheng

**Affiliations:** ^1^Department of Laboratory Medicine, Nanfang Hospital, Southern Medical University, Guangzhou, China; ^2^Nanfang Hospital, Southern Medical University, Guangzhou, China; ^3^Institute of Antibody Engineering, School of Laboratory Medicine and Biotechnology, Southern Medical University, Guangzhou, China

**Keywords:** alternative splicing, splicing factors, cervical cancer, prognosis, survival

## Abstract

**Background:**

Increasing evidence suggests that aberrant alternative splicing (AS) events are associated with progression of cancer. This study evaluated the prognostic value and clarify the role of AS events in cervical cancer (CC).

**Methods:**

Based on RNA-seq AS event data and clinical information of CC patients in The Cancer Genome Atlas (TCGA) database, we sought to identify prognosis-related AS events in this setting. We selected several survival-associated AS events to construct a prognostic predictor for CC through the least absolute shrinkage and selection operator (LASSO) and multivariate Cox regression. Moreover, Kyoto Encyclopedia of Genes and Genomes and Gene Ontology analyses were performed on genes with prognosis-related AS events and constructed an AS-splicing factors (SFs) regulatory network.

**Results:**

2770 AS events were significantly correlated with overall survival (OS). The area under the curve (AUC) values of receiver-operator characteristic curve (ROC) for the final prognostic predictor were 0.926, 0.946 and 0.902 at 3, 5, and 10 years, respectively. These values indicated efficiency in prognostic risk stratification for patients with CC. The final prognostic predictor was an independent predictor of OS (HR: 1.24; 95% CI: 1.020–1.504; *P* < 0.05). The AS-SFs correlation network may reveal an underlying regulatory mechanism of AS events.

**Conclusion:**

AS events are essential participants in the prognosis of CC and hold great potentials for the prognostic stratification and development of treatment strategy.

## Introduction

Mounting evidence shows that abnormal gene expression is closely related to the genesis and progression of tumors. Various studies have been focused on differences in gene expression to discover potential diagnostic, prognostic biomarkers and therapeutic targets in tumors (Weiskirchen, 2016). Although some promising results have been found for cancers, most of those studies only concentrated on gene expression levels, ignoring the transcriptional structure regulated by alternative splicing (AS). AS plays a major role in post-transcriptional regulation (Ge and Porse, 2014). More than 90% of human genes are modified by AS (Nilsen and Graveley, 2010) that regulates gene expression (Wagner and Berglund, 2014) produces transcript variants (Kelemen et al., 2013; Climente-Gonzalez et al., 2017) and increases protein diversity (Yang et al., 2016). Research has shown that aberrant AS is closely related to various diseases, including cancer (Kim et al., 2018; Wong A. C. H. et al., 2018a). Through the in-depth genomic and functional studies, AS abnormalities and generated specific subtypes have been identified as driving factors of tumors (Oltean and Bates, 2014; Todaro et al., 2014). Furthermore, AS events involving oncogenic processes have been reported, including angiogenesis, metastasis, proliferation, apoptosis, and invasion (David and Manley, 2010; Porazinski and Ladomery, 2018). Notably, AS events are mainly regulated by splicing factors (SFs), whose mutations (Salton et al., 2015) or changes in expression may cause AS abnormalities (Shilo et al., 2014; Sveen et al., 2016) and activation of oncogenes and tumorigenic pathways (Shilo et al., 2014; Koh et al., 2015; Salton et al., 2015; Dvinge et al., 2016). Hence, AS events or SFs could be invoked as potential diagnostic or prognostic targets for cancer.

Cervical cancer (CC) is one of the most common malignant tumors in gynecology, and its morbidity and mortality rank fourth in female malignant tumors (Bray et al., 2018). Some studies have reported that the incidence ages of cervical cancer patients tended toward younger (Schoell et al., 1999). Screening and diagnostic methods for cervical cancer have been gradually improved, and great progress has been achieved in surgical treatment and radio-chemotherapy. However, the long-term survival rate and quality of life of patients have yet to be improved. Once pelvic lymph node metastasis occurs, the 5-year overall survival (OS) rates for patients with early stage cervical cancer decrease to 53% (Liu et al., 2015). Studying the genesis and development mechanism of CC at the molecular level is conducive to discovering new molecular targets and providing a research basis for the targeted and precise treatment of CC. Therefore, it is necessary to further investigate the molecular mechanism of prognosis in patients with cervical cancer CC. Numerous studies have shown that dysregulation of AS events and cancer-specific AS events could be used as potential diagnostic or prognostic biomarkers and even treatment targets for cancer (Miura et al., 2012; Le et al., 2015; Chen et al., 2019). However, there are few studies focusing on the clinical significance and potential regulatory mechanism of AS in CC. According to the types of splicing, AS events are divided into seven types, namely Retained Intron (RI), Mutually Exclusive Exons (ME), Alternate Promoter (AP), Alternate Terminator (AT), Alternate Acceptor site (AA), Alternate Donor site (AD), and Exon Skip (ES) (Li et al., 2017). In addition, AS is mainly regulated by SFs, whose mutation or changes in expression are closely related to tumors. AS abnormalities can lead to some diseases (including cancer), which may be related to SFs. Therefore, it is necessary to investigate the potential regulatory relationship of AS-SFs (Kedzierska and Piekielko-Witkowska, 2017; Ratnadiwakara et al., 2018), which may be helpful in clarifying the pathogenesis of CC and providing new diagnostic and treatment directions.

The Cancer Genome Atlas (TCGA) database contains rich and complete AS patterns, RNA-seq data and patient clinical information. We conducted a systematic and comprehensive analysis of CC-AS data and constructed an AS prognostic model for patients with CC. We established an AS-SFs regulatory network and clarified the role of AS events as prognostic biomarkers for CC. This study provides novel insight into the diagnosis and treatment of CC.

## Materials and Methods

### Data Extraction and Pre-processing

**FIGURE 1:**
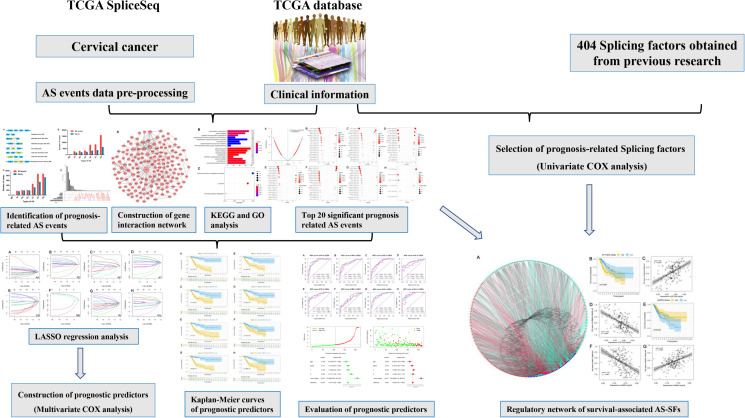
Flowchart of the whole research.

RNA-seq data and clinical information of the TCGA-CC cohort were obtained from the TCGA database^1^. RNA-seq AS events data for CC cohort were available at TCGA SpliceSeq database^2^. A total of 253 cancer samples [Cervical Adenosquamous Carcinoma (*n* = 4), Cervical Endometrioid Carcinoma (*n* = 2), Cervical Squamous Cell Carcinoma (*n* = 214), Endocervical Adenocarcinoma (*n* = 26), Mucinous Carcinoma (*n* = 7)] and three normal samples. In the TCGA database, a total of 307 samples with clinical follow-up information. In this study, quantitative analysis and comparison of the seven types of AS events was performed using percent-spliced-in (PSI) values (ranging from zero to one). Percentage of samples with PSI value >75% were downloaded, and we further processed Percentage of samples with PSI value ≤30% and minimum PSI standard deviation ≤0.01 were deleted. Figure 1 shows the flowchart of the whole study.

### Survival Analysis

In this study, CC patients with OS ≥90 days were selected. Follow-up time ranged from 90 to 4,086 days. We performed univariate Cox regression analysis to evaluate the connection between the PSI values of each AS event and OS (*P* < 0.05), and assess the relationship between the expression of each SFs gene and OS in the TCGA CC (*P* < 0.05).

### Functional Enrichment Analysis

We used the R package “clusterProfiler” to perform functional annotations for survival-associated AS event genes and investigate the relevance of these dysfunctional genes, and visualized via Cytoscape software (version 3.7.1). Both *P*-adjust and *q*-value >0.05 denoted statistical significance in the Gene Ontology and the Kyoto Encyclopedia of Genes and Genomes terms analysis.

### Construction of the Prognostic Predictor Model

The top 20 prognosis-associated AS events in the seven types were selected as candidates and further filtered using LASSO regression analysis. Subsequently, we used multivariate Cox regression to construct prognostic predictors models for patients with CC. Univariate and multivariate Cox regression analyses were performed on the following clinical factors to assess whether the final prognostic predictor was an independent predictor of OS in CC: age (≥50/<50), histologic grade (G3–G4/G1–G2), clinical stage (Stage III–IV/Stage I–II), tumor stage, tumor status, and final prognostic predictor (high risk/low risk). The standard deviation (SD) of risk score was calculated, and the result (risk Score/SD) of each sample was included as a new continuous variable for Cox regression analysis.

### Potential Correlation Network of Survival-Associated AS-SFs

A total of 404 SFs have been reported in previous studies (Seiler et al., 2018). Expression data of 404 SFs in CC were obtained from the TCGA database. Subsequently, univariate Cox regression analysis was performed to identify survival-related SFs. Prognosis-related SFs expression values and AS events PSI values were used to construct an AS-SFs correlation network based on the following: the absolute value of the Pearson correlation coefficient was >0.1 and *P* < 0.05. The AS-SFs correlation network plot was visualized using the Cytoscape software (version 3.7.1).

### Statistical Analysis

All statistical analyses were performed using the R software (version 3.6.1). The intersection plot between genes and seven types of AS events was visualized via the UpSetR package in R software. The prognostic predictor model value was assessed through the survivalROC package in R software. Kaplan-Meier curves and Pearson correlation analyses were performed using the “survival” and “basicTrendline” packages in R software. LASSO regression analysis was performed using the “glmne” and “survival” packages in R software.

## Results

### Overview of Clinical Characteristics and AS Events in the TCGA-CC Cohort

**FIGURE 2:**
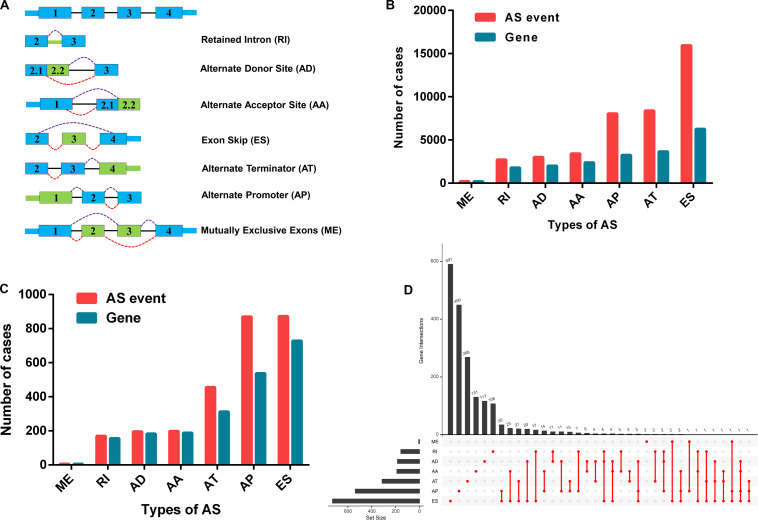
Prognosis-associated alternative splicing (AS) events. **(A)** Sketch map for the seven types of AS events in this research. **(B)** The number of AS events and associated genes in the TCGA CC cohort. **(C)** The number of prognosis-associated AS events and involved genes from the 307 patients with CC. **(D)** The UpSet plot showed the interactions between the seven types of survival-related AS events in CC. One gene may have up to four types of AS events related to patient prognosis.

We extracted alternative mRNA splicing events and clinical data for 307 patients with CC from the TCGA. The clinical features are presented in Table 1. Firstly, we performed univariate Cox regression analysis to evaluate the connection between clinical characteristics and outcome in the TCGA-CC (Table 1). Next, we investigated the prognostic value of AS events. There were seven types of AS events shown in Figure 2A. In the TCGA-CC cohort, we detected 41,776 AS events from 19,615 genes: 3,424 AAs in 2,398 genes, 3,017 ADs in 2,016 genes, 8,066 APs in 3,258 genes, 8,395 ATs in 3,664 genes, 15,942 ESs in 6,277 genes, 209 MEs in 202 genes, and 2,723 RIs in 1,800 genes (Figure 2B).

**TABLE 1:** Baseline data of TCGA CC patients.

Clinical parameters		Total (*N*)	OS	
			Hazard rations (95% CI)	*P* value
ALL		307	–	–
Age	Age ≥50	125	1.327 (0.830–2.121)	0.237
	Age <50	182		
Histologic grade	G3-G4	121	0.934 (0.552–1.578)	0.798
	G1-G2	154		
	NA	32		
Clinical stage	Stage III–IV	67	2.362 (1.440–3.874)	0.001
	Stage I/II	233		
	NA	7		
Tumor stage	T3-T4	31	3.672 (1.930–6.985)	7.39E-05
	T1-T2	213		
	NA	63		
Distant metastasis	M1	10	3.494 (1.171–10.428)	0.0249
	M0	166		
	NA	181		
Lymph node stage	N1	60	2.766 (1.392–5.495)	0.004
	N0	135		
	NA	112		
Tumor status	With tumor	74	20.037 (10.663–37.650)	1.225E-20
	Tumor free	191		
	NA	42		

*OS, overall survival; CI, confidence interval; NA, not available. Tumor status: Person neoplasm status (tumor free, with tumor).*

### Survival-Related AS Events in the TCGA-CC Cohort

AS events significantly associated with OS were obtained by performing univariate Cox regression analysis in the TCGA-CC cohort. A total of 2,770 AS events in 2,115 genes were identified as prognosis-associated AS events: 873 ESs in 729 genes, 870 APs in 538 genes, 456 ATs in 313 genes, 199 AAs in 189 genes, 196 ADs in 184 genes, 170 RIs in 156 genes, and six MEs in six genes (Figure 2C and Supplementary Table S1). Therefore, one single gene may have multiple types of AS events that are associated with prognosis. ES events were the most commonly observed pattern in AS events, followed by AP and AT events. In addition, the UpSet plot illustrated that individual genes have multiple prognosis-associated AS events (Figure 2D).

### Functional Enrichment Analysis of Genes With Survival-Associated AS Events in CC

**FIGURE 3:**
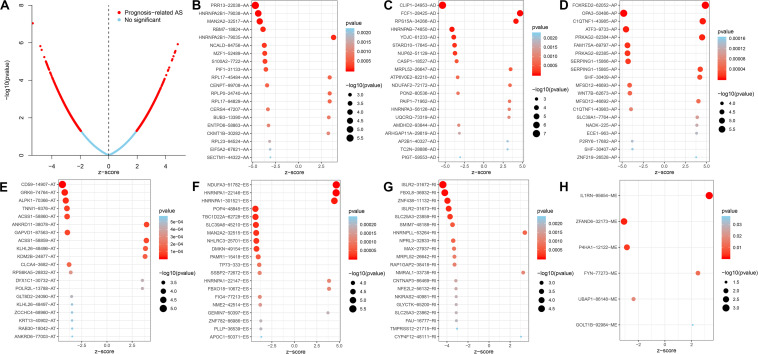
Top 20 of significant prognosis related AS events in the CC cohort. **(A)** Red dots indicate survival-related AS events in CC. Blue dots indicate AS events unrelated to survival in CC. **(B–H)** Bubble plots of the top 20 survival associated AA, AD, AP, AT, and RI events in CC, respectively.

**FIGURE 4:**
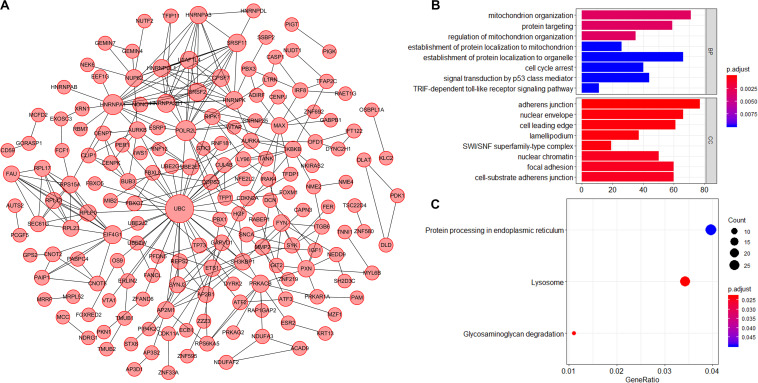
Molecular characteristics of genes with survival-associated AS events in CC. **(A)** Gene interaction network of genes with survival-associated AS events in CC generated by Cytoscape. Larger circles indicate greater importance. **(B)** GO analysis of genes with prognosis-related AS events. BP: Biological process; CC: Cellular component. **(C)** KEGG pathway analysis of genes with prognosis-related AS events.

AS events significantly associated with prognosis are shown in Figure 3A. The top 20 significant prognosis-related AS events are presented in Figures 3B–H. Among those, only six ME events were related to prognosis (Figure 3H). Subsequently, we performed bioinformatics analyses to investigate the molecular characteristics of genes with prognosis-related AS events. As shown in Figure 4A, genes significantly correlated with survival (*P* < 0.005) were selected to generate gene interaction networks using the Cytoscape software (version 3.7.1). Ubiquitin C (*UBC*), heterogeneous nuclear ribonucleoprotein A1 (*HNRNPA1*), and RNA polymerase II subunit L (*POLR2L*) were the major hub genes identified in the networks. Gene ontology (GO) analysis found that “mitochondrion organization,” “protein targeting,” “regulation of mitochondrion organization,” “establishment of protein localization to mitochondrion,” “establishment of protein localization to organelle,” “cell cycle arrest,” “signal transduction by p53 class mediator” and “*TRIF*-dependent toll-like receptor signaling pathway” were the most significant biological process terms; “adherens junction,” “nuclear envelope” and “cell leading edge” were the three most significant cellular component terms (Figure 4B). Kyoto Encyclopedia of Genes and Genomes (KEGG) pathway analysis revealed that these genes were mainly involved in the “protein processing in endoplasmic reticulum” and “lysosome” pathways (Figure 4C).

### Construction of Prognostic Predictors Based on AS Events in Patients With CC

**FIGURE 5:**
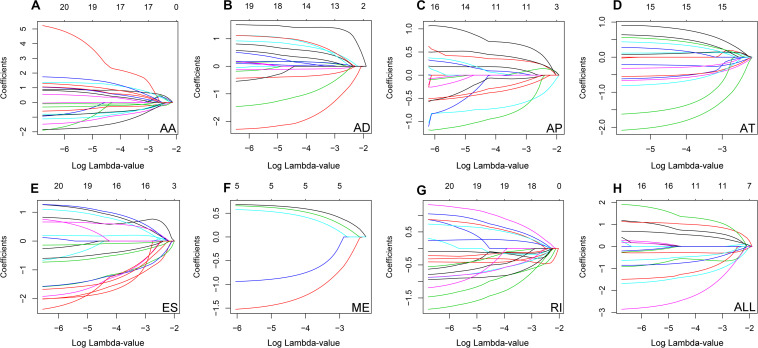
Candidate AS events was filtrated based on LASSO regression analysis.

**FIGURE 6:**
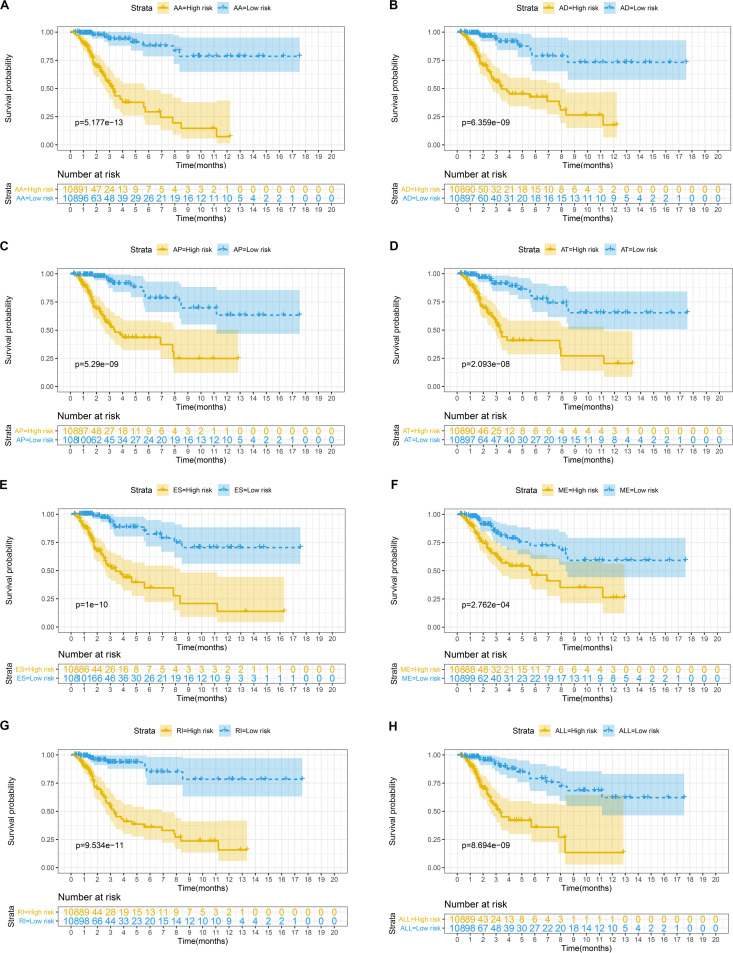
**(A–G)** Kaplan-Meier curves of prognostic predictor constructed with seven types of AS events in CC patients, respectively. The yellow line indicates a high-risk group; the blue line indicates a low-risk group. **(H)** Kaplan-Meier curves of the final prognostic predictor built with all survival associated candidates AS events in the seven types in CC patients. The yellow line indicates a high-risk group; the blue line indicates a low-risk group.

**FIGURE 7:**
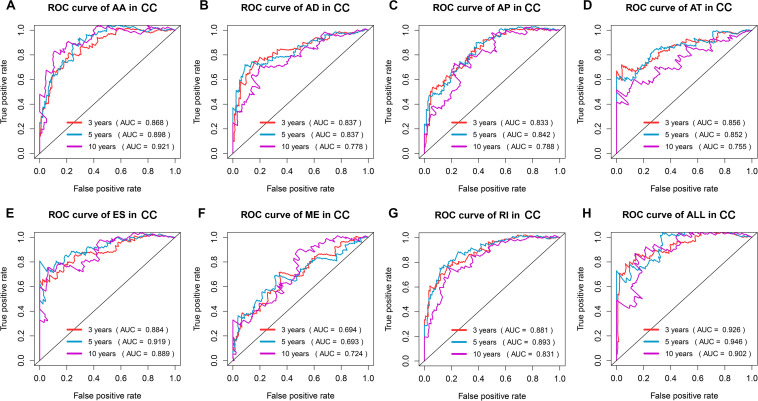
ROC curves with AUCs of prognostic predictor constructed with single type or seven types of AS events in CC patients, respectively. Red line represents 3 years, cyan line represents 5 years, and purple line represents 10 years.

**FIGURE 8:**
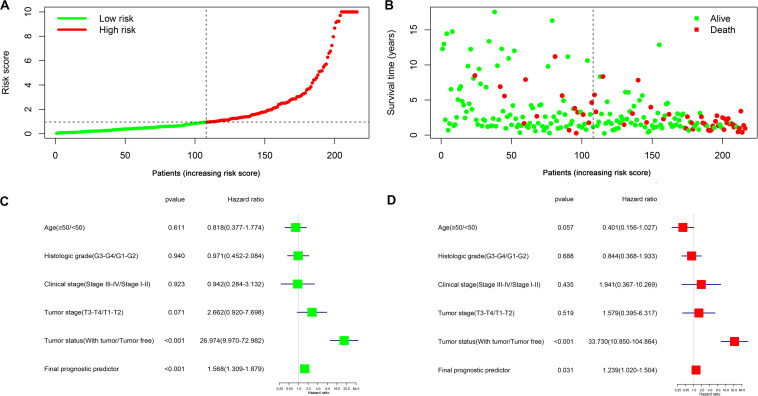
Identification capability of prognostic predictor for classifying CC patients into high and low risk groups. **(A)** The distribution of risk score for CC patients; high-risk (red) and low-risk (green). **(B)** Scatter plot shows the survival status and survival time of CC patients. Red dots denote patients that are dead and green dots denote patients that are alive. **(C)** Univariate Cox regression analysis. Forest plot of the association between risk factors and survival of CC patients. **(D)** Multivariate Cox regression analysis. The final prognostic predictor was an independent predictor of prognosis in in CC.

The top 20 prognosis-associated AS events in the seven types were selected as candidates and further filtered through the least absolute shrinkage and selection operator (LASSO) regression analysis. Subsequently, survival-related AS events in the seven types were selected to establish prognostic predictors via multivariate Cox regression (Figure 5 and Table 2). In addition, all prognosis-associated candidate AS events in the seven patterns were merged to construct the final prognostic predictor. The results demonstrated that eight prognostic prediction models can predict the clinical outcome of patients with CC (Figures 6A–H). We plotted the receiver-operator characteristic (ROC) curves and calculated areas under the curve (AUCs) to compare the efficiency of these predictors. We found that the AUC values of the final prognostic predictor model were >0.9 at 3, 5, and 10 years; these values were better than those obtained from other models constructed with single types of AS events (Figures 7A–H). Hence, the final prognostic predictor was the best prognostic predictor model (Figure 8A). This predictor can well stratify the prognosis of CC patients (Figure 8B). Univariate analysis showed that tumor status and the final prognostic predictor were significantly correlated with OS in CC (Figure 8C). Furthermore, multivariate Cox regression analysis showed that the final prognostic predictor was significantly associated with prognosis in CC (HR: 1.24; 95% CI: 1.020–1.504; *P* < 0.05) (Figure 8D). The above results indicated that the final prognostic predictor was an independent predictor of OS in patients with CC. Eight CC specific AS events involved in the final prognostic predictor are listed in Table 3.

**TABLE 2:** Prognostic predictors for CC patients.

Splice type	Formula (Gene-As id-Splice type)	Hazard ratio (95% CI)	AUC		
			3 years	5 years	10 years
AA	HNRNPA2B1-79035-AA*1.226 + RPL17-45484-AA*2.757 + BUB3-13390-AA*1.624 + RPL23-94524-AA*(-1.155) + ZNF18-39297-AA*(-1.716) + LY6K-85356-AA*0.851 + FUT3-46948-AA*(-1.151) + MIEF2-39602-AA*0.950 + ZFP64-59815-AA*2.066 + NAT6-64991-AA*(-1.267) + ANO8-48279-AA*0.943	3.702 (1.106–13.429)	0.868	0.898	0.921
AD	FCF1-28425-AD*1.571 + YDJC-61233-AD*(-2.902) + UQCRQ-73319-AD*0.928 + AP2B1-40327-AD*0.878 + PIGT-59553-AD*(-0.457) + BPTF-43116-AD*(-1.350) + MIB2-198-AD*1.275 + SLC38A1-21328-AD*1.247	2.219 (0.905–5.635)	0.837	0.837	0.778
AP	FOXRED2-62052-AP*0.944 + SERPING1-15866-AP*(-0.838) + SHF-30409-AP*0.560 + WNT7B-62673-AP*(-1.119) + P2RY6-17682-AP*(-0.490) + GEMIN4-38259-AP*(-0.507)	1.061 (0.537–2.210)	0.833	0.842	0.788
AT	PTCHD4-76445-AT*1.051 + C1orf86-247-AT*(-2.410) + IGF1-24050-AT*(-0.798) + ST8SIA1-20727-AT*(-0.891) + C4orf36-69840-AT*(-1.687) + USP6NL-10751-AT*0.725 + HGF-80248-AT*0.718 + ERLIN2-83348-AT*(-1.326)	1.047 (0.515–2.208)	0.856	0.852	0.755
ES	MAN2A2-32515-ES*(-2.333) + NHLRC3-25701-ES*(-1.522) + NME2-42514-ES*(-1.342) + ZNF782-86986-ES* (-2.048) + EBPL-25914-ES*1.428 + TATDN1-85097-ES*(-2.090) + ERBB2IP-72262-ES*(-0.704) + PFDN5-22007-ES*1.335 + MUC4-68197-ES*(-2.346) + HNRNPA2B1-79037-ES*1.193 + REPIN1-82248-ES*(-0.578) + PACRGL- 68904-ES*(-1.646)	1.120 (0.480–2.724)	0.884	0.919	0.889
ME	IL1RN-95654-ME*0.698 + P4HA1-12122-ME*(-1.568) + FYN-77273-ME*0.678 + UBAP1-86148-ME*(-0.966) + GOLT1B-92984-ME*0.603	1.280 (0.735–2.275)	0.694	0.693	0.724
RI	ZNF438-11132-RI*(-1.248) + HNRNPLL-53264-RI*1.131 + MAX-27937-RI*(-0.595) + MRPL52-26642-RI*(-2.091) + NMRAL1-33738-RI*1.143 + CNTNAP3-86469-RI*(-0.801) + GLYCTK-65200-RI*(-1.068) + CYP4F12-48111-RI *0.775 + CCDC74B-55280-RI*0.718 + RPL13-38089-RI*(-1.114)	1.315 (0.701–2.564)	0.881	0.893	0.831
ALL	FOXRED2-62052-AP*0.874 + FCF1-28425-AD*1.394 + NDUFA3-51782-ES*1.614 + MAN2A2-32517-AA*(-1.127) + NHLRC3-25701-ES*(-1.602) + SHF-30409-AP*0.583 + WNT7B-62673-AP*(-1.948) + RBM7-18824-AA*(-2.983)	1.745 (0.699–4.539)	0.926	0.946	0.902

**TABLE 3:** CC specific AS events involved in the final prognostic predictor.

Gene	As id	Splice type	Exons	Hazard ratio (95% CI)	*P*-value
FOXRED2	62052	AP	1.1	6.866 (3.155–14.941)	1.20E-06
FCF1	28425	AD	1.2	11.933 (4.235–33.622)	2.72E-06
NDUFA3	51782	ES	4.3	8.787 (3.524–21.911)	3.13E-06
MAN2A2	32517	AA	17.1	0.150 (0.062–0.360)	2.21E-05
NHLRC3	25701	ES	4	0.103 (0.036–0.294)	2.29E-05
SHF	30409	AP	3	2.377 (1.586–3.563)	2.74E-05
WNT7B	62673	AP	1	0.117 (0.042–0.329)	4.60E-05
RBM7	18824	AA	4.1	0.089 (0.026–0.302)	1.05E-04

### Potential Correlation Network of Survival-Associated AS-SFs in CC

**FIGURE 9:**
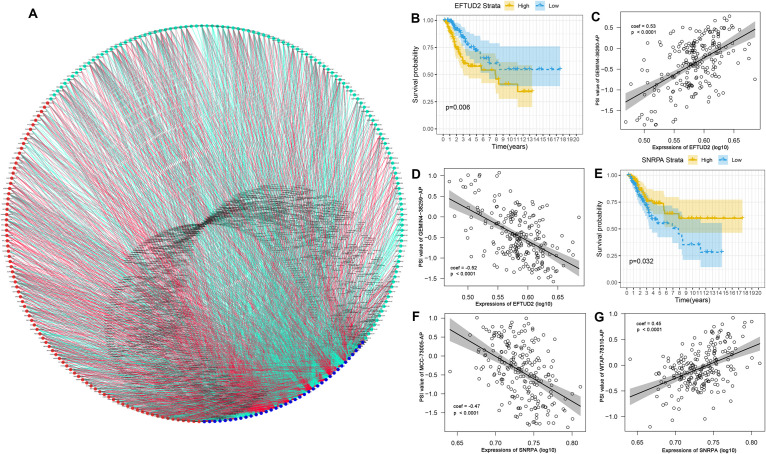
Correlation network of survival-associated SFs-AS in CC. **(A)** Correlation network between expression values of survival-related SFs and PSI values of survival-related AS, generated by Cytoscape. Blue dots indicate survival-related SFs. Cyan/Red dots indicate favorable prognostic/poor prognostic AS events, respectively. Red/cyan lines indicate positive/negative correlations between SFs and AS events. **(B)** Splicing factor *EFTUD2* was related to prognosis in CC. The yellow line indicates a high expression group; the Blue line indicates a low expression group. **(C)** High expression of *EFTUD2* was positively correlated to PSI values of *GEMIN4-38260-AP*. **(D)** High expression *EFTUD2* was negatively correlated to PSI values of GEMIN4-38259-AP. **(E)** Splicing factor *SNRPA* was related to prognosis in CC. The yellow line indicates a high expression group; the Blue line indicates a low expression group. **(F)** High expression of *SNRPA* was negatively correlated to PSI values of *MCC-73005-AP*. **(G)** High expression of *SNRPA* was positively with PSI values of *WTAP-78310-AP*.

SFs are the main factors regulating AS events (Carazo et al., 2019). They bind to pre-mRNAs and regulate the selection of splicing sites and exons. Therefore, it was essential to investigate the correlation network of AS-SFs. Univariate Cox regression analysis found that 36 SFs were related to survival in the TCGA-CC cohort (Supplementary Table S2). Next, we performed Pearson correlation coefficient analysis to characterize the connection between the expression of 36 survival-related SFs and percent-spliced-in (PSI) value of the 206 top significant survival-associated AS events (*P* < 0.001). The regulatory network is demonstrated in Figure 9A. The expression of 36 survival-associated SFs (blue dots) was significantly associated with the PSI value of 206 survival-associated AS events. Among them, 121 and 85 events were favorable (cyan dots) and poor (red dots) prognosis AS events. Remarkably, the results indicated that the majority of poor prognostic AS events (red dots) were positively correlated (red lines) with SFs, whereas the most of favorable prognostic AS events (cyan dots) were negatively correlated (cyan lines) with SFs. Representative correlations between SFs and specific AS events are shown in dot plots (Figures 9C,D,F,G). For example, SF small nuclear ribonucleoprotein polypeptide A (*SNRPA*) was linked to favorable prognosis, whereas elongation factor Tu GTP binding domain containing 2 (*EFTUD2*) was a poor prognostic SF in CC (Figures 9B,E). Correlation analysis indicated that high expression of *EFTUD2* was negatively correlated with *GEMIN4-38259-AP* (favorable prognostic AS event) (Supplementary Figure S1A) and positively correlated with *GEMIN4-38260-AP* (poor prognostic AS event) (Supplementary Figure S1B). Similarly, correlation analysis showed that high expression of *SNRPA* was positively correlated with *WTAP-78310-AP* (favorable prognostic AS event) (Supplementary Figure S1C) and negatively correlated with *MCC-73005-AP* (poor prognostic AS event) (Supplementary Figure S1D).

## Discussion

Gene expression and the diversity of the generated proteins are regulated by AS. Numerous diseases are associated with aberrant AS events, including the occurrence and progression of tumors. In human cancer, various forms of AS exist and various cancer-related genes are regulated by AS. In tumor cells, AS events abnormalities generate protein diversity, which promotes tumor cell proliferation and metastasis (Liu and Cheng, 2013; Oltean and Bates, 2014). In this study, we systematically and comprehensively analyzed AS events in CC, and performed functional enrichment analysis for genes with prognosis-related AS events. Eight AS events were selected to establish an ideal prognostic model for patients with CC. Moreover, an AS-SFs regulatory network was constructed to clarify the pathogenesis and provide new ideas for the diagnosis and treatment of CC.

Although cytology combined with virus screening can reduce the morbidity of cervical cancer, the quality of life and long-term survival rate of patients require further improvement. Currently, the prognostic factors of cervical cancer are not fully understood. Studies have reported the prognostic factors of cervical cancer including age, clinical stage, tumor size, histological type, differentiation degree, lymph node metastasis, surgical margins, invasion depth, and intravascular tumor thrombus (Sevin et al., 1996; Van de Putte et al., 2005). Some studies (Panek et al., 1999; Cheng and Xie, 2003) performed univariate and multivariate regression analyses on these prognosis-related pathological parameters, with inconsistent results. The discovery of reliable prognostic indicators of cervical cancer can avoid undertreatment and overtreatment. However, in terms of the overall research status, studies investigating the prognosis of cervical cancer were mainly focused on clinicopathology, while research on molecular biological indicators was relatively limited. Therefore, the identification of novel prognostic molecular markers for CC has important clinical significance, potentially providing a new direction for the clinical treatment of this disease and enhancing its prognosis.

Next-generation sequencing technology is advancing, laying the foundation for the investigation of aberrant AS patterns. Investigation of the AS events can offer a deeper understanding of the molecular mechanism of tumorigenesis and progression and provide a new direction for the development of tumor markers. Some studies have reported that AS events can be used as prognostic indicators for tumors, such as papillary thyroid cancer (Lin et al., 2019), colorectal cancer (Zong et al., 2018), ovarian cancer (Zhu et al., 2018), gastrointestinal cancer (Lin et al., 2018), and hepatocellular carcinoma (Chen et al., 2019). Klaes et al. (1999), reported *TSG101* variants were abnormally expressed during the progression of cervical neoplasia. The above studies suggested that AS events may be used as prognostic markers for CC.

However, there are few studies focusing on AS events in CC. In this study, we found that 2,770 AS events were significantly associated with OS. AS events were affected by its pre-mRNA, thus, we performed bioinformatics analysis on genes with AS events. In the weighted network diagram, *UBC, HNRNPA1* and *POLR2L* were the major hubs genes. Previous studies have shown that *UBC* and *HNRNPA1* participated in the biological process of CC. Studies have shown that ubiquitin-specific protease 7 could promote cervical carcinogenesis (Su et al., 2018), while *HNRNPA1* was a good diagnostic marker for cervical cancer (Kim et al., 2017). Functional enrichment analysis found that these genes were mainly involved on the mitochondrion and lysosome-related pathways. Xia et al. (2019) investigated canolol induced apoptosis in Hela cells via the mitochondrial signaling pathway. Takeda et al. (2019) found that insulin like growth factor 2 receptor (*IGF2R*) disorders could cause lysosome dysfunction and regulate the apoptosis of cervical cancer cells. Furthermore, mitochondria and lysosomes interact with each other during cellular activity and regulate apoptosis. Wong et al. also reported the functional correlation between mitochondrial and lysosomal dysfunction (Wong Y. C. et al., 2018b). The above results supported the accuracy and reliability of our bioinformatics analysis. We hypothesized that these survival-related AS events may cause mitochondrial or lysosomal dysfunction, affecting the progression of cervical cancer.

In this study, we found that AS events can be used for the prognostic stratification of patients with CC. Moreover, it was shown that a gene can generate multiple mRNAs through AS, some of which exert an opposite effect. For example, *GEMIN4-38259-AP* and *WTAP-78310-AP* are favorable prognostic factors, whereas *GEMIN4-38260-AP* and *WTAP-78311-AP* are poor prognostic factors for patients with CC (Supplementary Figure S1). The same phenomenon has been observed in previous studies showing that AS of the *ZAK* gene generated two subtypes (*ZAK*α and *ZAK*β), which perform antagonistic functions (Lee et al., 2018). Similarly, AS generates two isoforms of *BCL2L1* gene (*BCL-XL* and *BCL-XS*); *BCL-XS* promotes apoptosis, whereas *BCL-XL* inhibits apoptosis (Chen and Weiss, 2015). LASSO regression is suitable for the analysis of high-dimensional data (Tibshirani, 1997). Hence, we filter a list of AS events using LASSO regression analysis to construct prognostic prediction models. We constructed an ideal prognostic prediction model with AUC values of ROC >0.9 at 3, 5, and 10 years, indicating efficiency for the prognostic risk stratification of patients with CC. To the best of our knowledge, in the CC AS events studies, this was the first study to integrate AS events and clinical characteristics, and subsequently perform univariate and multivariate Cox regression analyses to comprehensively analyze the prognostic value of AS events in patients with CC. Univariate and multivariate cox regression analyses showed that the final prognostic predictor was significantly correlated with OS. The final prognostic predictor was an independent prognostic indicator for patients with CC.

SFs are the main factors regulating AS events (Seiler et al., 2018). In this study, we also investigated the potential role of SFs. We selected survival-related SFs and AS events to construct an AS-SFs regulatory network for clarifying the pathogenic mechanism of CC. We found that most of the favorable prognosis AS events were negatively correlated with the expression of SFs, whereas the majority of poor prognosis AS events were positively correlated with the expression of SFs. These findings are similar to the conclusions reported in previous studies (Lin et al., 2019). However, whether the upregulation of some specific SFs can increase the number of poor prognosis AS events and reduce that of favorable prognosis AS events requires further investigation. To our knowledge, in the CC AS events studies, this was the first to explore the combination of AS and SFs, which may help to clarify the potential mechanism of AS in the oncogenesis of CC and provide new ideas for the diagnosis and treatment of CC. However, our study also has some limitations, namely the lack of an independent cohort verification and functional experiments to further explore the function of abnormal AS events and SFs on tumorigenesis and development. Our future research will focus on these parts.

Repeated and persistent high-risk human papillomavirus (HR-HPV) infection is the main initiating factor of cervical cancer, among which the most important types are HPV16 and 18 (Hong, 2010). The HR-HPV early genes *E6* and *E7* are the main initiating factors of cervical cancer, but not enough to cause cancer alone (Ghittoni et al., 2010). AS and SFs play an important role in tumors. HPV, AS and SFs are involved in the occurrence and development of cervical cancer. Liu et al. (2018) found that HR-HPV oncoproteins E6 and E7 increase the expression of Splicing factor *SRSF10* via *E2F1* transcriptional activation. *SRSF10* mediated *IL1RAP* alternative splicing regulates cervical cancer oncogenesis. Splicing factors also affect HPV gene expression. During HPV infection, splicing factor *hnRNP1* overexpression can cause HPV16 late gene expression (Somberg et al., 2009). Meanwhile, splicing factors *SRSF1* (Somberg and Schwartz, 2010), *SRSF3* and *SRSF9* (Jia et al., 2009) can be combined with the splicing enhancer region downstream of the splicing site (*SA3358*) of the HPV16 genome to participate in the regulation of early and late HPV16 gene expression. HPV16 genome also produces variable alternative mRNA splicing through the splice sites *SD226* and *SA409*, generate a variety of HPVl6 *E6* and *E7* oncoproteins (Stacey et al., 1995). It can be seen that after HPV infection, the HPV genome will variable mRNA splicing and produce a variety of oncoproteins. The early genes of HPV will affect SFs, which can mediate the gene AS events, involved in the development and prognosis of cervical cancer. In this study, there are few samples for HPV testing, and the data is not available [HPV18/16 (*n* = 15), Other HPV type(s) (*n* = 7), NA (*n* = 285)]. This is also the inadequacy of this paper.

In conclusion, we constructed a good prognostic prediction model for CC, established the prognostic value of AS events and SFs, explored the potential regulatory mechanism, and clarified the potential function of AS in the occurrence of CC. These findings may offer new possible directions for the diagnosis and treatment of CC. Meanwhile, these survival-related AS events and SFs provide numerous valuable targets for verification in the future.

## Data Availability Statement

The datasets (TCGA-CESC cohort) for this study can be found in the TCGA database, https://portal.gdc.cancer.gov/projects. The datasets (RNA-seq AS events data) for this study can be found in the TCGA SpliceSeq database, https://bioinformatics.mdanderson.org/TCGASpliceSeq/.

## Author Contributions

LZ and YW: conceptualization and supervision. XS and HZ: data curation. XS and JD: formal analysis and visualization. LZ: funding acquisition. All authors: investigation, project administration, and manuscript review and editing. XS, JD, and HZ: validation. XS: writing – original draft.

## Conflict of Interest

The authors declare that the research was conducted in the absence of any commercial or financial relationships that could be construed as a potential conflict of interest.

## Footnotes

1 https://portal.gdc.cancer.gov/projects

2 https://bioinformatics.mdanderson.org/TCGASpliceSeq/

## Supplementary Material

FIGURE S1 **(A–E)** Kaplan-Meier curves for AS events *GEMIN4-38259-AP, GEMIN4-38260-AP, WTAP-78310-AP, MCC-73005-AP* and *WTAP-78311-AP* with high (yellow line) and low (blue line) PSI value group in CC, respectively.

TABLE S1 Survival related AS events were filtered by univariate COX regression.

TABLE S2 Survival-related SFs were filtered by univariate COX regression.
