# Supplementary figures and images for: Prognostic Value and Potential Role of Alternative mRNA Splicing Events in Cervical Cancer
Source: Front Genet. 2020 Jul 10;11:726. doi: 10.3389/fgene.2020.00726 (PMC7394696; doi:10.3389/fgene.2020.00726)

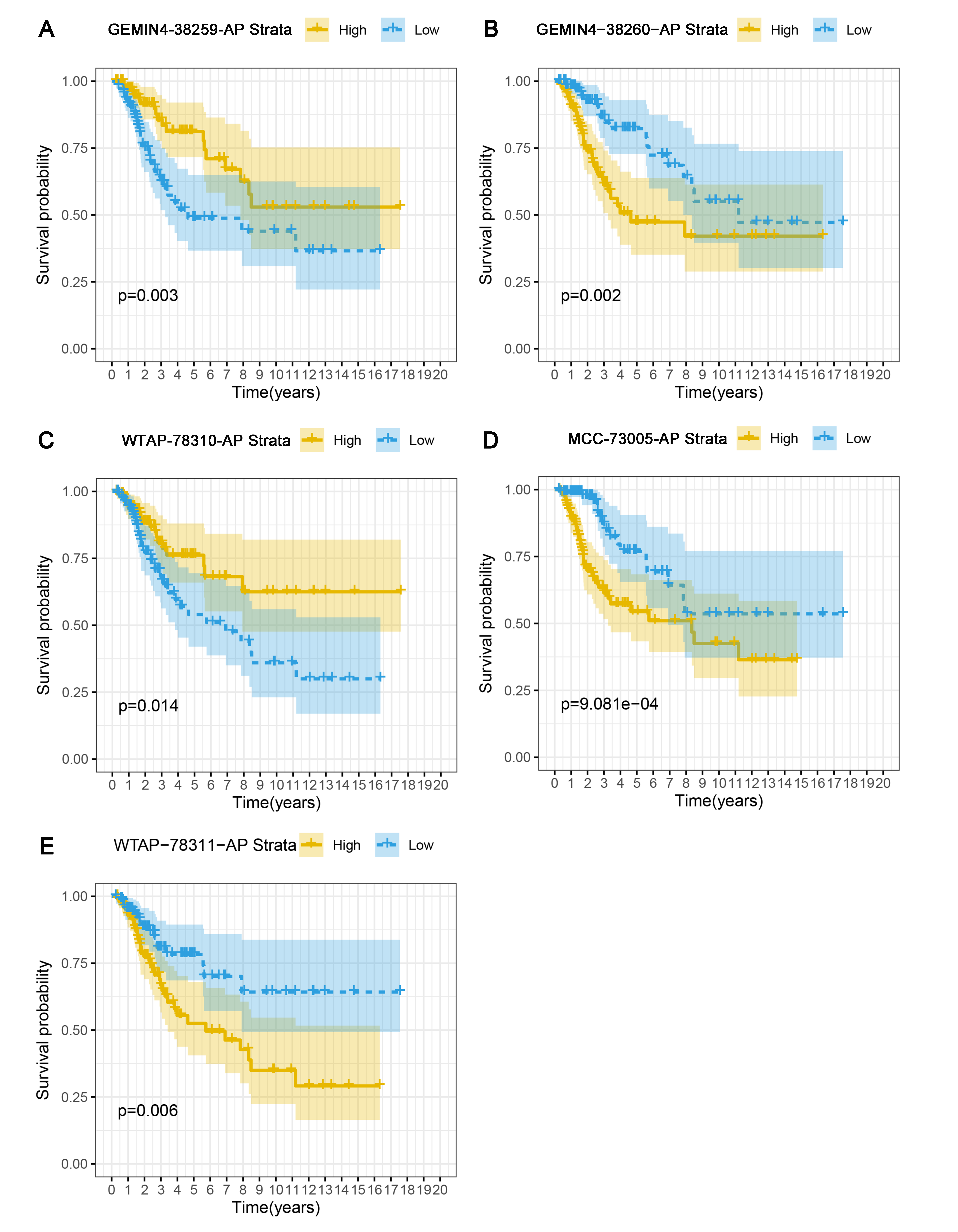

Supplement: FIGURE S1 — (A–E) Kaplan-Meier curves for AS events GEMIN4-38259-AP, GEMIN4-38260-AP, WTAP-78310-AP, MCC-73005-AP and WTAP-78311-AP with high (yellow line) and low (blue line) PSI value group in CC, respectively. [file Image_1.TIF]
