# Supplementary material for: Prognostic Value and Potential Role of Alternative mRNA Splicing Events in Cervical Cancer
Source: Front Genet. 2020 Jul 10;11:726. doi: 10.3389/fgene.2020.00726 (PMC7394696; doi:10.3389/fgene.2020.00726)
Supplement: TABLE S2 — Survival-related SFs were filtered by univariate COX regression. [file Table_2.DOCX]

**Supplementary Table 2. Survival-related SFs were filtered by univariate COX regression.**

| Splicing factors | HR | HR.95L | HR.95H | p-value |
| --- | --- | --- | --- | --- |
| FMR1 | 0.41340508 | 0.232317603 | 0.735647052 | 0.002664296 |
| DDX1 | 2.76623251 | 1.413025156 | 5.415361691 | 0.002990592 |
| PRPF40B | 2.948783214 | 1.421857912 | 6.115465106 | 0.00366447 |
| RBM3 | 0.499445535 | 0.312616808 | 0.797928441 | 0.003680891 |
| DNAJC6 | 2.032464071 | 1.253286796 | 3.296061375 | 0.004037652 |
| PQBP1 | 0.413485057 | 0.226387135 | 0.755210283 | 0.004059799 |
| C9orf78 | 0.316537511 | 0.144095671 | 0.695343553 | 0.004171425 |
| HSPA5 | 1.970603503 | 1.234341634 | 3.146031907 | 0.004482305 |
| FAM50A | 0.483590402 | 0.286130907 | 0.817317079 | 0.006660237 |
| ZC3H13 | 2.244061444 | 1.247735621 | 4.035960567 | 0.006954202 |
| GPKOW | 0.446910774 | 0.238539745 | 0.837299627 | 0.011926228 |
| SNRPA | 0.377071699 | 0.174670217 | 0.814008643 | 0.01298848 |
| CWC22 | 2.594458944 | 1.222709398 | 5.505165188 | 0.012998783 |
| PCBP3 | 1.559279829 | 1.094018857 | 2.222405554 | 0.014011853 |
| GEMIN5 | 2.32978095 | 1.186330024 | 4.575353541 | 0.014043015 |
| SNRNP25 | 0.508518377 | 0.295093498 | 0.876301717 | 0.014870313 |
| CXorf56 | 0.50174928 | 0.283032802 | 0.889481142 | 0.018231157 |
| EFTUD2 | 2.522220452 | 1.163898781 | 5.465763958 | 0.019046805 |
| DDX18 | 2.34826244 | 1.129763224 | 4.880966532 | 0.02220781 |
| TOP1MT | 1.593602855 | 1.061076232 | 2.393390768 | 0.024726196 |
| SF3A2 | 0.539453636 | 0.313875317 | 0.92715231 | 0.025502592 |
| PRMT5 | 1.57937001 | 1.054292989 | 2.365954867 | 0.02666683 |
| ZC3H11A | 2.185895595 | 1.085827147 | 4.400460576 | 0.028479177 |
| PPIE | 2.012272382 | 1.072691356 | 3.774841772 | 0.029362623 |
| SNRPE | 2.118487964 | 1.076542217 | 4.168894803 | 0.029742392 |
| PSIP1 | 0.619248744 | 0.39924805 | 0.960478095 | 0.032352624 |
| INTS3 | 1.803665877 | 1.046211996 | 3.109513758 | 0.03379286 |
| SNRPD3 | 0.48029085 | 0.243581137 | 0.947032694 | 0.034254074 |
| RBM4 | 2.57073099 | 1.072540087 | 6.161688408 | 0.034261683 |
| CLK2 | 1.991994691 | 1.042178766 | 3.807449338 | 0.03707326 |
| SRPK2 | 1.777024355 | 1.031450396 | 3.061529251 | 0.038308757 |
| WBP4 | 1.995126875 | 1.033673005 | 3.85086118 | 0.03952542 |
| DDX39A | 0.561054188 | 0.323300533 | 0.973650736 | 0.039887943 |
| ISY1 | 0.580854968 | 0.344512461 | 0.979333211 | 0.041518001 |
| SNRPN | 0.774922933 | 0.60597705 | 0.990970783 | 0.042128092 |
| CCDC12 | 0.616156386 | 0.381096486 | 0.996200977 | 0.048212408 |
